# Supplementary material for: Pharmacodynamics and Outcomes of a De-Escalation Strategy with Half-Dose Prasugrel or Ticagrelor in East Asians Patients with Acute Coronary Syndrome: Results from HOPE-TAILOR Trial
Source: J Clin Med. 2021 Jun 18;10(12):2699. doi: 10.3390/jcm10122699 (PMC8234416; doi:10.3390/jcm10122699)
Supplement: Supplementary file 1 [file jcm-10-02699-s001.zip › jcm-1254338-supplementary.pdf]

Supplementary data

Table S1. Timing of platelet function testing by VerifyNow.

Timing	Overall (n=120)	Prasugrel (n=39)	Ticagrelor (n=40)	Clopidogrel (n =41)	*p*-Value
At 1 month					0.457
9:00-12:00	87 (72.5)	27 (69.2)	32 (80.0)	28 (68.3)	
13:30-16:30	33 (27.5)	12 (30.8)	8 (20.0)	13 (31.7)	
At 3 months					0.718
9:00-12:00	68 (56.7)	20 (51.3)	24 (60.0)	24 (58.5)	
13:30-16:30	52 (43.3)	19 (48.7)	16 (40.0)	17 (41.5)	

The data are presented as number (%).

Table S2. Platelet reactivity measured by VerifyNow according to sampling time.

Variables	9:00-12:00	13:30-16:30	*p*-Value
At 1 month PRU			
Prasugrel	9 (5-94)	48 (6-62)	0.843
Ticagrelor	7 (3-30)	7 (4.5-64.5)	0.409
Clopidogrel	151 (71-219)	183 (159-206)	0.203
At 3 months PRU			
Prasugrel	82 (31-145)	84 (47-138)	0.744
Ticagrelor	15 (6-52)	14 (5-44)	0.748
Clopidogrel	115 (77-166)	196 (166-224)	0.006

The data are presented as median (interquartile range).

Table S3. Platelet reactivity status of oral P2Y12 inhibitors at chronic time points.

Variables	Prasugrel	Ticagrelor	Clopidogrel	*p*-Value
At 1 month				<0.001
HPR (208)	0 (0)	0 (0)	10 (24.4)	
OPR (85-208)	8 (20.5)	0 (0)	23 (56.1)	
LPR (85)	31 (79.5)	40 (100.0)	8 (19.5)	
At 3 months				<0.001
HPR (208)	0 (0)	0 (0)	8 (19.5)	
OPR (85-208)	19 (48.7)	5 (12.5)	26 (63.4)	
LPR (85)	20 (51.3)	35 (87.5)	7 (17.1)	

Data are presented as number (%). HPR, high platelet reactivity; LPR, low platelet reactivity; OPR, optimal platelet reactivity.

Table S4. The occurrence of BARC type 1 and 2 bleedings.

Outcomes	Overall (n=120)	Prasugrel (n=39)	Ticagrelor (n=40)	Clopidogrel (n =41)	*p*-Value
9-month bleeding	30 (25.0)	12 (30.8)	13 (32.5)	5 (12.2)	0.057
BARC type 1	27 (22.5)	11 (28.2)	11 (27.5)	5 (12.2)	
BARC type 2	3 (2.5)	1 (2.6)	2 (5.0)	0 (0)	
Landmark analysis at 30 days					NA
Before 30 days bleeding BARC type 1	8 (6.7)	5 (12.8)	1 (2.5)	2 (4.9)	0.170
Beyond 30 days bleeding	22 (19.6)	7 (20.6)	12 (30.8)	3 (7.7)	0.035
BARC type 1	19 (17.0)	6 (17.6)	10 (25.6)	3 (7.7)	
BARC type 2	3 (2.7)	1 (2.9)	2 (5.1)	0 (0)	

Data are presented as number (%). BARC, Bleeding Academic Research Consortium.

Table S5. The efficacy outcomes at 9 months clinical follow-ups.

Outcomes	Overall (n=120)	Prasugrel (n=39)	Ticagrelor (n=40)	Clopidogrel (n =41)	*p*-Value
MACCE	2 (1.7)	0 (0)	1 (2.5)	1 (2.4)	NA
Cardiac death	0 (0)	0 (0)	0 (0)	0 (0)	-
MI	1 (0.8)	0 (0)	1 (2.5)	0 (0)	-
TVR	2 (1.7)	0 (0)	1 (2.5)	1 (2.4)	NA
Stroke	0 (0)	0 (0)	0 (0)	0 (0)	-

Data are presented as number (%). MACCE, major adverse cardiac and cerebrovascular events; MI, myocardial infarction; TVR, target vessel revascularization. NA, not applicable.

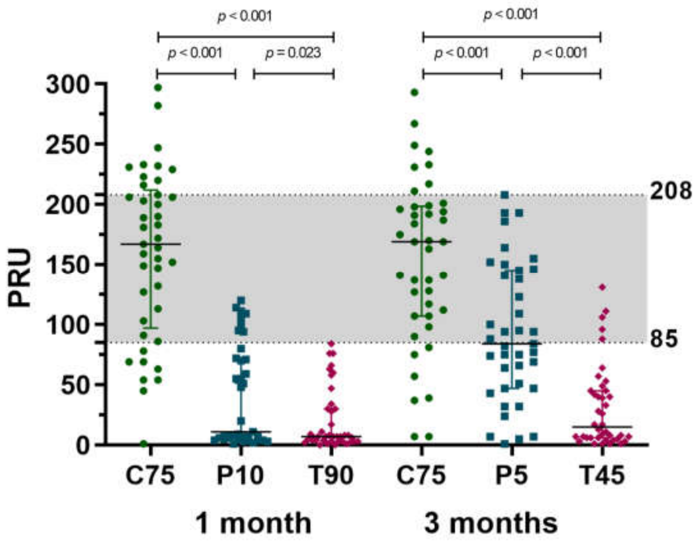

Figure S1. Pharmcodynamic comparisons between P2Y12 inhibitors at chronic time points in accordance with the VerifyNow P2Y12 method at 1 and 3 months. Scatter plots representing P2Y12 reaction unit (PRU) results for study groups. Gray area indicates optimal platelet reactivity (OPR, defined as PRU values between 85 and 208).
